# Renal effects of environmental and occupational lead exposure

**DOI:** 10.4103/0019-5278.44689

**Published:** 2008-12

**Authors:** S. K. Rastogi

**Affiliations:** CSIR Emeritus Scientist (Former Deputy Director and Head Epidemiological Section), Indian Institute of Toxicology Research, Post Box No. 80, Mahatma Gandhi Marg, Lucknow - 226 001, India. E-mail: subodhkrastogi@gmail.com

Lead is one of the most useful elements in industry, but serves no useful function in the human body. Environmental and industrial lead exposures continue to pose major public health problems in the exposed population.[1] Over the years, it has become increasingly evident that low-level lead exposure resulting in blood lead levels between 10 and 15 μg/dL can lead to deleterious effects like cognitive impairment and behavioral deficits, high blood pressure (BP) and impaired renal function.[2,3] Lancereaux[4] provided the first description of kidney disease and interstitial nephritis by postmortem examination of a lead-poisoned artist. It was not until the late 1920s when an epidemic of chronic nephritis in Queensland, Australia, was linked to childhood lead poisoning that the full spectrum of lead-induced nephropathy became apparent.[5,6] This was followed by cases of renal diseases from the US in individuals consuming lead-contaminated illegally distilled moonshine whisky.[7]

## ENVIRONMENTAL LEAD EXPOSURE

Environmental lead exposure continues to be a public health problem. In the past, lead-based paint was a major source of lead poisoning among children. The painted surfaces of old houses contained significant amounts of lead. Direct ingestion of lead paint, lead-contaminated house dust and water by children has been identified as a major contributor to lead poisoning among the children. Many studies have confirmed that lead-contaminated dust is a major determinant of lead concentrations in blood.[8] Similarly, a highly significant correlation between lead concentration in drinking water and blood lead concentrations has been reported.[9] Children are more susceptible to the effects of environmental lead than adults because of the increased gastrointestinal absorption of lead in children. Children are more vulnerable because they absorb lead 5–10 times more effectively than adults and have a greater exposure because of their exploratory behavior and frequent hand to mouth activity.[10] Adults at the highest risk are those exposed to lead fumes or dust in the industry.[11,12]

## OCCUPATIONAL LEAD NEPHROPATHY

An association between lead poisoning and renal diseases in humans has been recognized and documented by several studies.[8,12,13] Elemental lead and inorganic lead compounds are absorbed by ingestion or inhalation, but organic lead compounds, e.g. tetraethyl lead, may also be absorbed by skin contact. Organic lead compounds are the most toxic. Absorption of lead from the lungs is very efficient, especially if the particles are less than 1 μm in diameter. The gastrointestinal absorption of lead varies with the age of the individual; children absorb around 50% of what they ingest but adults only absorb 10–20% of what they ingest. Lead is very similar to calcium chemically. Thus, once in the body, it is handled as if it were calcium. Lead serves no useful purpose in the human body and its presence in the body can lead to toxic effects, regardless of the exposure pathway.

The kidney is the critical organ after long-term occupational or environmental exposure to lead. Excessive exposure to lead may cause acute or chronic nephrotoxic effects. Two types of nephropathy, acute and chronic nephropathy, have been observed in humans. Acute Pb nephropathy is characterized functionally by a generalized deficit of tubular transport mechanisms (Fanconi syndrome) and morphologically by the appearance of degenerative changes in the tubular epithelium and the nuclear inclusion bodies containing Pb protein complexes.[15,16] These effects, which are usually reversible with chelation therapy, have been reported mainly in children manifested by glycosuria and aminoaciduria. Chronic occupational exposure to lead has also been linked to a high incidence of renal dysfunction, which is characterized by glomerular and tubulointerstitial changes, resulting in chronic renal failure, hypertension and hyperuricemia. Chronic lead nephropathy is an irreversible renal disease that develops over months or years of excessive exposure.[17,18] This has been reported in adults who had ingested leaded paint during childhood and those who consumed illicitly distilled alcohol (moonshine whisky).[14,15] In the chronically exposed adults, Pb nephropathy occurs as a progressive tubulointerstitial nephritis that is difficult to diagnose at the early stage. Incipient Pb nephropathy is not associated with urine abnormalities easily detected by dipsticks. The tests evaluating the glomerular filtration rate (GFR) (creatinine clearance, blood urea nitrogen, serum creatinine) are the only ones that can be used to detect the renal effect caused by the occupational exposure to Pb.[14,19,20] But, when these tests are abnormal, the nephropathy has already reached the irreversible phase that may lead to renal sufficiency.[19] Chronic low-level exposure to lead is also associated with an increased urinary excretion of low molecular weight proteins and lysosomal enzymes.[21] Epidemiologic studies have shown an association between blood lead levels and BP, and hypertension is a cardinal feature of lead nephropathy.[13,20,22,24]

Most lead-associated renal effects or toxicity are a result of the ongoing chronic or current high acute exposure. They can also be attributable to a previous chronic lead exposure. The lowest level at which Pb has an adverse effect on the kidney remains unknown. Both glomerular and tubular effects have been reported.[20] The glomerular effects range from an increased prevalence of high molecular weight proteinuria to a nephrotic syndrome.[18,23] The reported tubular changes consist of an enhanced urinary excretion of enzymes.

## BIOMARKERS OF NEPHROTOXICITY

The prevention of renal diseases induced by exposure to industrial or environmental Pb largely relies on the capability to detect nephrotoxic effects at a stage when they are still reversible or at least not yet compromising the renal function. During the past decade, various tests have been proposed for the early detection of the toxic effects at different sites on the nephron. Some of these tests have been validated and some need epidemiological validation.

Currently, there are some early and sensitive indicators available that are considered predictive or indicative of renal toxicity from lead exposure. Recent studies have shown more than 20 potential markers of renal effects that can be arbitrarily classified into three broad categories.[25–28] Table 1 shows the different biomarkers used in Pb-induced nephrotoxicity.

**Table 1:** Showing the different biomarkers used in Pb-induced nephrotoxicity.

No.	Reference	Exposure	Exposure duration (years)	B Pb conc. (μg/dL)	Biomarkers used
1	Lilis *et al*.[25]	Occupational	> 10	79	GFR, SCr
2	Cramer *et al*.[26]	Occupational	9	103	GFR, HP
3	Wedeen *et al*.[27]	Occupational	5	48	GFR, HP, TMPAH
4	Wedeen *et al*.[28]	Occupational	14	51	GFR, HP
5	Hong *et al*.[29]	Occupational	7	68	GFR, TMG
6	Lilis *et al*.[30]	Occupational	12	80	SCr, BUN
7	Dekort *et al*.[31]	Occupational	12	47	SCr, BUN
8	Verschoor *et al*.[33]	Occupational	< 2–10	47	UNAG, URBP
9	Staeseen *et al*.[34]	Environmental	NA	10	SCr
10	Omae *et al*.[35]	Occupational	0.1–26	37	CCr, CUA, Uβ2μG, Cβ2μG
11	Hu[36]	Environmental	NA	6	Ccr
12	Kim *et al*.[37]	NA	NA	10	Scr
13	Fels *et al*.[38]	Environmental	NA	13	SCr, UE, UP, ULMWP
14	Hsiao *et al*.[39]	Occupational	13	40	SCr
15	Sonmez *et al*.[40]	Occupational	0.14	25	UNAG
16	Muntner *et al*.[41]	NA	NA	5	SCr

B Pb conc., blood lead concentration; NA, not available; GFR, glomerular filtration rate; SCr, serum creatinine; HP, histopathology; TMPAH, transport max. for PAH; TMG, transport max for glucose; BUN, blood urea nitrogen; UNAG, urine N-acetyl-β-D-glucosaminidase; URPB, urine retinal binding protein; CCr, creatinine clearance; CUA, uric acid clearance; Uβ2μG, urine β2μG; UP, urine proteins; ULMWP, urinary low molecular weight proteins.

### Functional markers

1. Creatinine in serum (crt-S).
2. Creatinine in urine (crt-U).
3. Urinary proteins of low or high molecular weight.
  3.1.High molecular weight proteins – albumin, transferrin, immunoglobulin G.
  3.2.Low molecular weight proteins – retinol-binding protein (URBP), β_2_ -microglobulin (β_2_ -m), urinary α-1-microglobin (Uα1m).
4. Urinary enzymes – N-acetylglucosaminidase (NAG).
5. Alkaline phosphatase (ALP).
6. γ-glutamyl transferase (γ-GT).

These are the biological markers of tubular damage, which are characterized by enhanced urinary excretion of α-1-microglobulin, β_2-_ m, RBP, NAG, APL and γ-GT.

### Cytotoxicity markers

These include:

1. The brush border tubular antigens (BBA, BB_50_ and HF_5_).
2. Enzymes – B-galactosidase.

Exposed Pb workers show an increased leakage of tubular antigens and several enzymes as a sign of renal toxicity. This is in all likelihood a reflection of the damage to the proximal tubular cells.

### Biochemical markers

These include:

1. Eicosanoids – 6-keto-prostaglandin F_1_α (6-keto-PGF_1_α), prostaglandin F_2_α (PGF_2_α), prostaglandin E_2_(PGE_2_).
2. Thromboxane (TxB_2_).
3. Fibronactin.
4. Urinary sialic acid activity, sialic acid in plasma or in RBCs.
5. Urinary kallikrein activity.
6. Urinary glycosaminoglycans (GAG).
7. Intestinal alkaline phosphate (IAP).

The most outstanding effect found in the workers exposed to Pb is an interference with the renal synthesis of eicosanoids, resulting in a lower urinary excretion of 6-keto-PGFα and an enhanced excretion of TXB_2_. It is generally accepted that the urinary 6-keto-PGF_1_α and TXB_2_ primarily reflect the glomerular synthesis of prostacyclin and TXA_2_, whereas urinary PGE_2_ and PGF_2_α are largely contributed by the renal medulla. The decrease in PGE_2_, PGF_2_α and enhanced excretion of TXB_2_ resulting from biochemical or cytotoxic effects in the medulla and glomeruli represent the earliest renal changes associated with exposure to Pb.[29,30]

The early effects on urinary excretion of 6-keto-PGFα and TXB_2_ suggest that the initial insult in Pb nephropathy might also involve the vasculature and glomeruli and is not exclusively localized in the tubulointerstitial compartment. The changes in the renal synthesis of eicosanoids raises the question of their relevance to health and are indicative of the degenerative process that may lead to a loss of renal function.[32]

Together with the changes in the urinary excretion of eicosanoids, the increased excretion of Tamm–Horsfall glycoprotein (THG) appears as an early renal effect induced by exposure to Pb. This increase could reflect an injury to the epithelial cells of the ascending limb of the Loop of Henle and the most proximal part of the distal convoluted tubules where this glycoprotein is localized. The physiological function of THG is still obverse. It might have several important functions, such as rendering the ascending limb of Henley's loop impermeable to water, transport to sodium, defense against infection or the immuneregulation of several cytokines.[22,32]

Urinary kallikrein is a serine proteases synthesized by the distal tubular cells, which might serve as an index of distal nephrotoxicity. As most of the kallikreine is associated with the membranes that face the urinary compartment, an increased urinary excretion of kallikrein could result from toxic damage in the distal tubular cells.

The increased urinary excretion of sialic acids appears as a rather early effect of exposure to lead. The GAGs are polysacchrides composed of repetitive disaccharide units. They are found in the glomeruli and the tubules and their leakage into the urine has been suggested to be a marker of injury to the nephron. An increased excretion of GAG has also been suggested to be an indicator of damage to the renal papilla, which is rich in GAG.[24]

## GLOMERULAR FILTRATION RATE

Creatinine clearance, blood urea nitrogen (BUN) and serum creatinine are some of the parameters that can be used to detect the renal effects caused by occupational exposure to Pb. But, when these tests are found abnormal, the nephropathy has already reached the irreversible phase that may lead to renal insufficiency.[17] The renal effects of Pb, consisting mainly in a decline of the GFR without proteinuria, have been reported in workers with a longstanding exposure to Pb, with a Pb-B of 600 μg/L or more.[20] So far, studies conducted on populations of workers with a lower level of exposure to Pb have disclosed no renal effect or only infraclinical changes of marginal significance.[12,13] In humans, a reduced GFR (i.e. indicated by decreases in the creatinine clearance or increases in the serum creatinine concentration) has been observed in association with exposures resulting in average PbBs < 20ug/dL. However, some studies have shown an increased GFR with Pb exposure. This may represent hyperfiltration, which may contribute to adverse renal effects. Decrements in GFR may contribute to an elevation in the BP, and an elevated BP may predispose people to glomerular disease. These effects may be mechanistically related and, furthermore, can be confounders and covariables in epidemiological studies.[28–30]
